# Dislocations of the Finger

**Published:** 1898-10-01

**Authors:** 


### DISLOCATIONS OF THE FINGER.

Dr. John M. Longford, house surgeon, Tynemouth Victoria Jubilee Infirmary, writes: Dr. R. H. Shaw's case of compound dislocation of ungual phalanx of thumb which you referred on September 17th reminds me of a similar case in a boy aged 16 who attended here last July with a dislocation at the first inter-phalangeal joint of the little finger. Although the tendons were not injured, there was an extensive wound through which the articular ends of the phalanges were protruding. I reduced the dislocation, sutured the wound, and dressed it antiseptically. Passive movements were begun after three days, and ten days after the accident the finger was restored almost absolutely to its normal condition.

